# Maternal Soluble Programmed Death Ligand-1 (sPD-L1) and T-regulatory Cells (Tregs) Alteration in Preeclampsia: A Cross-Sectional Study From Eastern India

**DOI:** 10.7759/cureus.67877

**Published:** 2024-08-26

**Authors:** Prakruti Dash, Saurav Nayak, Bharath Kumar Koppisetty

**Affiliations:** 1 Biochemistry, All India Institute of Medical Sciences, Bhubaneswar, Bhubaneswar, IND

**Keywords:** tgf-β1, high-sensitivity c-reactive protein (hscrp), interleukin (il)-6, spd-l1, regulatory t (treg) cells, early-onset preeclampsia

## Abstract

Background

Studies have shown that aberrant reactions of the immune system play an important role in the pathogenesis of preeclampsia. The immune checkpoint molecules programmed cell death protein 1/programmed death-ligand 1 (PD-1/PD-L1) system and the T-regulatory cells (Tregs) system are decisive in the regulation of immune responses and can be the target molecules in preeclampsia. In this study, an attempt has been made to evaluate the soluble PD-L1 (sPD-L1) in the serum of preeclampsia cases and correlate it with Tregs and inflammatory markers to have an insight into the link between these immunomodulatory molecules in the pathogenesis of preeclampsia.

Materials and methods

Ten normal fertile women, 20 trimester-matched normal pregnancy cases, and 20 preeclampsia cases were enrolled in the study. Serum sPD-L1, transforming growth factor beta 1 (TGF-β1), and IL-6 were measured by enzyme-linked immunosorbent assay (ELISA). High-sensitive C-reactive protein (hsCRP) was estimated using a clinical biochemistry autoanalyzer. Tregs were evaluated using flow cytometry.

Results and discussion

The immune checkpoint molecule PD-L1 inversely correlated with Tregs in preeclampsia cases. Associated inflammation was seen by raised IL-6 and hsCRP. The breakdown of immunological tolerance is mainly caused by the dysregulating the Tregs/Th17 balance, which leads to conditions of autoimmunity and chronic inflammatory disorders. PD-L1 can be the link between this immunological misbalance.

Conclusion

Our study, showing an increase in sPD-L1 and TGF and a decrease in Tregs with an increase in inflammatory markers like IL-6 and hsCRP levels in preeclampsia, has potential implications for early diagnosis and management of the condition. PD-L1 and Tregs can be target molecules for early management of preeclampsia.

## Introduction

Preeclampsia is a pregnancy-related multisystem disorder defined as the onset of hypertension during pregnancy characterized by a persistent high blood pressure of ≥140/90 and proteinuria of ≥300 mg/24 hours after 20 weeks of gestation in previously normotensive women. Preeclampsia is one of the prominent complications associated with pregnancy, resulting in increased incidence of maternal, fetal, and neonatal morbidity and mortality. It is estimated that in developing countries, preeclampsia accounts for 15% of maternal deaths every year. Management of preeclampsia is mostly centered around early delivery of the baby with a primary focus on control of blood pressure and prevention of seizures. The pathophysiology of the disorder is yet to be elucidated [1,2].

Programmed cell death protein 1 (PD-1) and programmed cell death ligand 1(PD-L1) are immune checkpoint systems imparting immunity to tumor cells. It is observed that tumor or cancer cells overexpress PD-L1, which interacts with PD-1 expressed on T cells, thus providing immunotolerance to the cancer cells. Antibodies against the PD-L1/PD-1 pathway have now been one of the immunotherapy modules against cancer [3]. Soluble PD-L1 (sPD-L1) and soluble PD-1 (sPD-1) are the soluble forms of membrane-bound PD-L1 and PD-1, generated by proteolytic cleavage of the membrane-bound forms and are detectable in the serum [4].

A fetus may be considered immunologically different from the mother as it expresses autogenic maternal antigens along with allogenic paternal antigens, but it remains protected from the maternal immune system. This can be partly explained by the PD-1/PD-L1 system. It is observed that PD-L1 is overtly expressed on the outer surface of syncytiotrophoblasts on chorionic villi in the placenta on its outer surface facing the maternal bloodstream in the uterus. Like cancer cells, it is expected to provide immunotolerance to the growing fetus [5].

T-regulatory cells (Tregs) are a particular subset of T lymphocytes CD4+, CD25+, and FOXP3+ that maintain immunological self-tolerance, suppress the inflammatory state, and induce immune homeostasis [6]. During pregnancy, Tregs are able to maintain immune tolerance by suppressing natural killer (NK) cells and T cell responses against allogeneic paternal antigens and self-antigens involved in rejection and complications associated with pregnancy. Studies have shown that Tregs count decreases in conditions like preeclampsia, whereas their number increases in normal pregnancy compared to non-pregnant fertile women [7].

Few observations depict that PD-L1/PD-1 and TGF-β1 stimulate the generation of Tregs and inhibit the formation of Th17. It thus increases the ratio between Tregs and Th17, thereby creating a niche in immunotolerance. This link between PD-L1/PD-1 and Tregs has been substantiated in a few studies done on pregnancy and preeclampsia [8]. A direct positive correlation has been documented between PD-L1/PD-1 and Tregs in pregnancy with increased counts of Tregs in normal pregnancy. In disorders like preeclampsia, dysregulation in the correlated pathway between the two immunoregulatory molecules has been shown in a few studies but has yet to be authenticated due to a lack of sufficient evidence-based data [9].

The inflammatory cytokines IL6 and high-sensitive C-reactive protein (hsCRP) modulate the expression of Tregs and are found to be increased in preeclampsia [7,10,11].

With the existing knowledge about PD-L1/PD-1 system and Tregs, this study was undertaken to find a link between sPD-L1 and Tregs in the serum in preeclampsia in order to assess the correlation between these two molecules and have an insight into the pathogenesis of preeclampsia from an immunological point of view. A link between sPD-L1 and Tregs, if seen, may help in early diagnosis and effective management targeting the pathological process undergoing preeclampsia.

## Materials and methods

This cross-sectional study was done in the Department of Biochemistry in association with the Department of Obstetrics and Gynaecology, All India Institute of Medical Sciences, Bhubaneswar, a tertiary care hospital in Eastern India.

The sample size was calculated to be 50 with an alpha error of 5% and power of 80%. The study was thus divided into three groups: group 1 comprised 10 non-pregnant fertile women; group 2 included 20 normal pregnant women; and group 3 consisted of 20 preeclampsia cases.

Group 1 had age-matched normal fertile non-pregnant women, and group 2 had both primiparous and multiparous healthy pregnant women with more than 20 weeks of gestation.

The preeclampsia group (group 3) comprised 20 women matching the diagnostic criteria of the International Society for the Study of Hypertension in Pregnancy (ISSHP) 2021:

i. Hypertension should be defined as a systolic BP (sBP) ≥ 140 mmHg and/or diastolic BP (dBP) ≥ 90 mmHg, based on an average of at least two measurements.

ii. Proteinuria should be defined as ≥ 30 mg/mmol urinary protein:creatinine ratio (PrCr) in a spot (random) urine sample, albumin:creatinine ratio (ACR) ≥ 8 mg/mmol, or ≥ 0.3 g/day in a complete 24-hour urine collection, or ≥ 2 + by urinary dipstick if confirmatory testing is not available.

The preeclampsia cases included were in the early preeclampsia group with a gestational age of less than 34 weeks.

The exclusion criteria were women with HELLP (hemolysis, elevated liver enzymes, and low platelets) syndrome, eclampsia, diabetes mellitus, other inflammatory disorders, and autoimmune disorders.

Informed written consent was obtained from all participants. The study was approved by the Institute Ethics Committee of All India Institute of Medical Sciences, Bhubaneswar.

Five milliliters of venous blood were taken from all participants; 3 mL was taken in ethylenediaminetetraacetic acid (EDTA) vial for flow cytometry, and 2 mL was taken in plain vials. Serum was extracted and used for enzyme-linked immunosorbent assay (ELISA) and automated chemistry analyzer.

Tregs were analyzed from whole blood using flow cytometry analysis using DURAClone IM Treg tubes by Beckman Coulter, Brea, CA. The DURAClone IM Treg panel enables the identification and characterization of FOXP3+ regulatory T cells using a fast permeabilization protocol. These tubes are a combination of eight markers in fluorochrome combinations that provide robust population identification, including CD3, CD4, CD25, CD39, CD45, CD45RA, FOXP3, and Helios. sPD-L1, TGF-β1, and IL-6 assays were performed using ELISA kits. hsCRP was estimated using an automated biochemistry analyzer.

All data were serialized and anonymized upon entry in Microsoft Excel (Microsoft® Corp., Redmond, WA). Appropriate statistical analysis was done. ANOVA was used to analyze the significance of tested parameters in the three groups. The correlation of PD-L1 levels with various anthropometric and measured biochemical and immunological parameters was done using Pearson’s correlation test. A p-value of less than 0.05 was considered statistically significant. All analyses were done using IBM SPSS version 26.0 (IBM Corp., Armonk, NY) and JASP version 0.17.2 (University of Amsterdam, Amsterdam, Netherlands).

## Results

CD4+FOXP3 was significantly lower in the preeclampsia group compared to normal pregnancy and non-pregnant fertile women. The count was highest in normal pregnant women. sPD-L1 level was highest in the preeclampsia group compared to groups 1 and 2, but it was not statistically significant. The inflammatory markers IL-6 and hsCRP were both raised significantly in preeclampsia cases compared to normal non-pregnant and normal pregnancy participants. Similarly, TGF-β1was significantly higher in the preeclampsia group compared to the other two study population groups. This has been tabulated in Table 1.

**Table 1:** The study parameters in the three groups Statistical significance based on the p-value of the one-way ANOVA test. CD - cluster differentiation; hsCRP - high-sensitive C-reactive protein; IL-6 - interleukin 6; sPD-L1 - soluble programmed cell death ligand 1; TGF-β1 - transforming growth factor beta 1

Parameter	Group 1, non-pregnant fertile (median (IQR))	Group 2, normal pregnancy (median (IQR))	Group 3, preeclampsia (median (IQR))	ANOVA, p-value
Weight (kg)	56.68 (48.25-60.12)	58.72 (55.27-61.01)	56.75 (52.32-59.87)	0.221
Systolic blood pressure (mmHg)	116 (110-122)	122 (119-125)	136 (130-142)	0.035
Diastolic blood pressure (mmHg)	55 (50-60)	62 (59-65)	82 (76-88)	0.011
Albumin (g/dL)	4.6 (4.2-4.6)	4.2 (4.0-4.4)	3.5 (3.1-3.6)	0.001
Lymphocytes %	26.61 (11.21-31.87)	8.50 (6.42-10.59)	11.54 (8.72-16.30)	0.002
Absolut count of lymphocytes	26,610.00 (11,207.50-31,870.00)	8,495.00 (6,425.00-10,587.50)	11,535.00 (8,720.00-16,302.50)	0.002
CD3+CD4+ %	34.61 (32.09-38.91)	33.80 (29.47-36.62)	29.80 (27.39-36.91)	0.292
CD4+CD25+ %	3.57 (2.83-4.05)	5.22 (3.49-7.16)	3.55 (1.92-5.74)	0.107
CD4+FOXP3+ %	3.70 (3.46-3.85)	4.80 (4.46-5.34)	1.46 (1.18-1.92)	<0.001
Absolute count of CD3+CD4+	11,549.36 (10,710.10-12,985.94)	11,277.39 (9,832.47-12,221.76)	9,942.59 (9,140.04-12,316.03)	0.292
Absolute count of CD4+CD25+	1,189.64 (944.37-1,350.65)	1,743.58 (1,163.78-2,389.29)	1,184.63 (641.54-1,915.44)	0.107
Absolute count of CD4+FOXP3+	1,234.69 (1,152.93-1,284.75)	1,601.76 (1,488.30-1,781.96)	488.87 (392.93-639.87)	<0.001
TGF-β1(pg/mL)	3,476.88 (2,443.06-4,836.06)	6,229.24 (3,089.54-6,753.06)	6,445.64 (5,336.97-6,663.34)	0.023
IL-6 (pg/mL)	16.39 (10.37-23.32)	12.40 (10.53-15.28)	35.19 (23.56-49.89)	0.001
hsCRP (mg/mL)	1.83 (1.12-2.33)	3.25 (2.33-5.04)	6.96 (3.48-26.07)	0.024
sPD-L1 (ng/mL)	0.47 (0.26-0.87)	0.60 (0.39-0.81)	0.62 (0.44-1.18)	0.152

sPD-L1 showed a positive and significant correlation with BP and inflammatory markers hsCRP and IL-6. However, TGF-β1 showed a non-significant positive correlation. There was a negative correlation of sPD-L1, Tregs, weight, and serum albumin. The correlation factors are shown in Table 2.

**Table 2:** The correlation of sPD-L1 levels with various anthropometric and measured biochemical and immunological parameters in the overall study population Statistical significance based on the p-value of Pearson’s correlation test. CD - cluster differentiation; hsCRP - high-sensitive C-reactive protein; IL-6 - interleukin 6; sPD-L1 - soluble programmed cell death ligand 1; TGF-β1 - transforming growth factor beta 1

Parameter	Pearson’s r	p-value
Weight (kg)	-0.381	0.006
Systolic BP	0.335	0.018
Diastolic BP	0.350	0.013
CD4+CD25+FOXP3	-0.329	0.026
TGF-β1	0.119	0.428
IL6	0.307	0.030
hsCRP	0.629	<0.001
Albumin	-0.449	0.001

**Figure 1:**
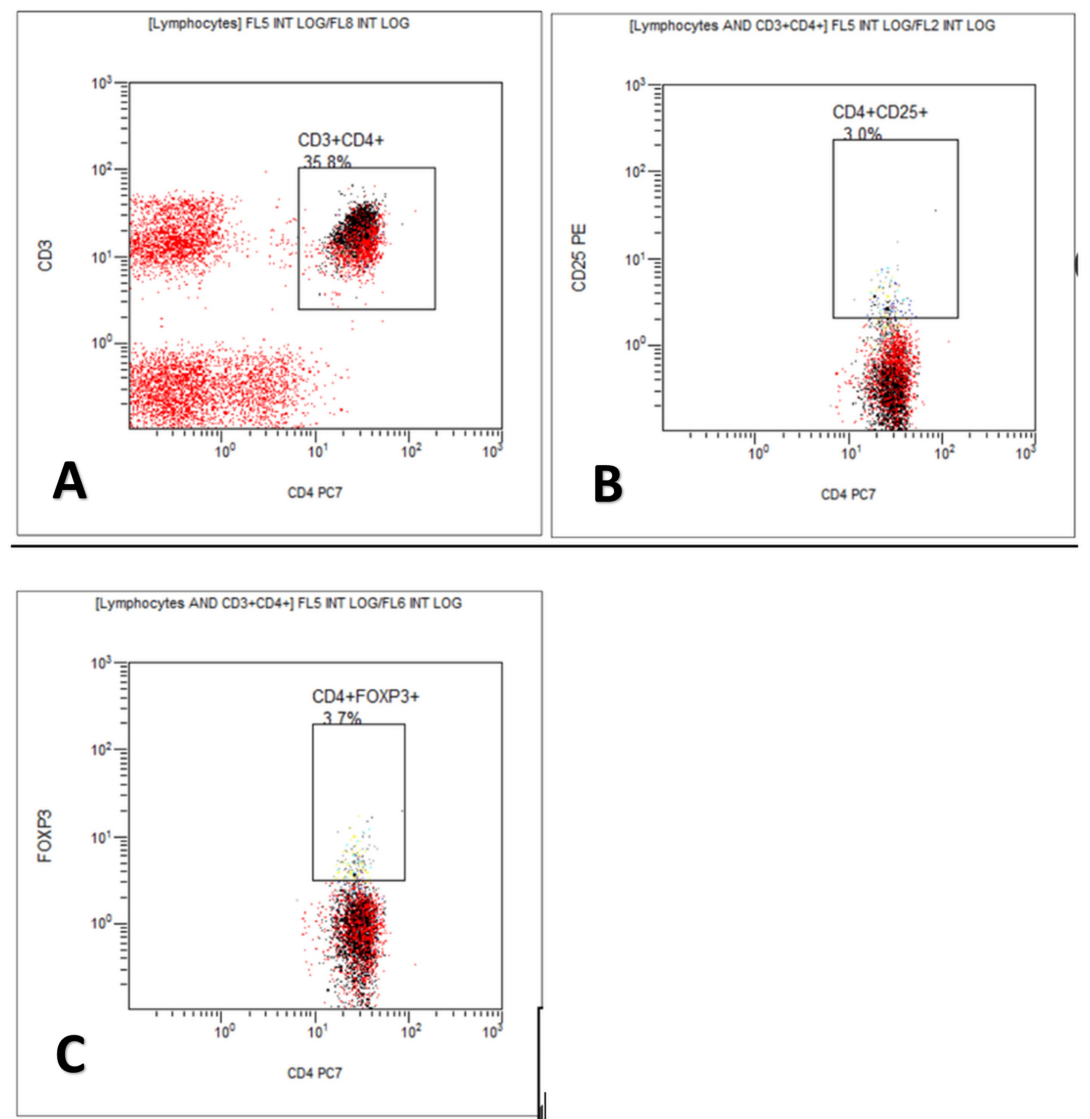
Flow cytometry flow page of T-regulatory cells in non-pregnant fertile women X-axis: in cell count; Y-axis: in cell count; A: flow cytometer count for CD3+CD4+cells; B: flow cytometer count for CD4+CD25+cells; C: flow cytometer count for CD4+FOXP3+cells

**Figure 2:**
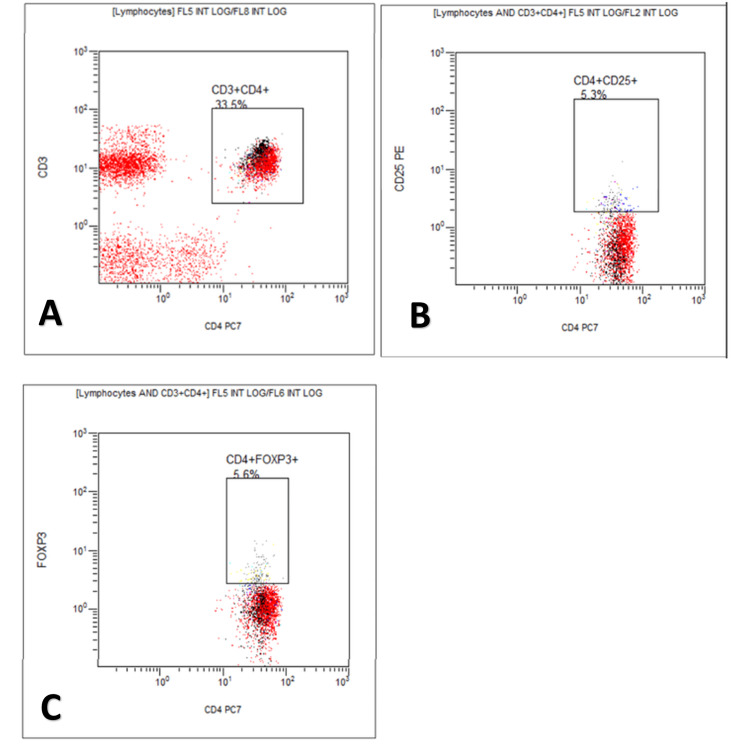
Flow cytometry flow page of T-regulatory cells in normal pregnancy X-axis: in cell count; Y-axis: in cell count; A: flow cytometer count for CD3+CD4+cells; B: flow cytometer count for CD4+CD25+cells; C: flow cytometer count for CD4+FOXP3+cells

**Figure 3:**
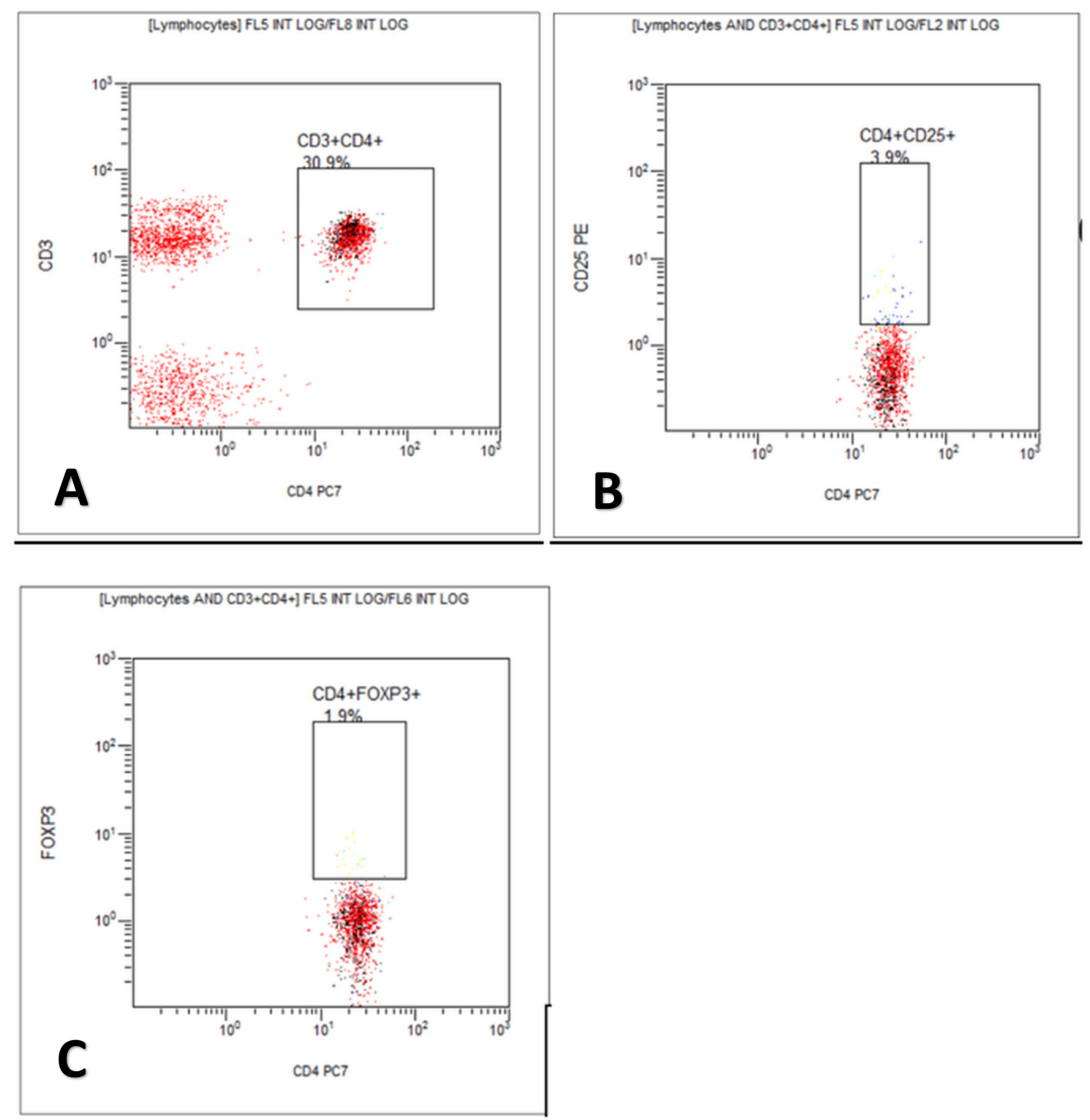
Flow cytometry flow page of T-regulatory cells in preeclampsia cases X-axis: in cell count; Y-axis: in cell count; A: flow cytometer count for CD3+CD4+cells; B: flow cytometer count for CD4+CD25+cells; C: flow cytometer count for CD4+FOXP3+cells

Figure 1, Figure 2, and Figure 3 show the flow cytometry flow page of CD3+CD4+, CD4+CD25+, and CD4+FOXP3+Tregs in the three study groups in the percentage of lymphocytes. Between the non-pregnant fertile group and preeclampsia, there is a significant difference in lymphocytes and CD4+FOXP3+Tregs. Between normal pregnancy and preeclampsia, there is a significant difference only in CD4+FOXP3+Tregs.

## Discussion

PD-L1/PD-1 comprises the receptor-ligand pathway responsible for providing immunotolerance in the body and has been considered for its role in cancer cell tolerance and escape from immune attack. The cancer cells evade the body’s immune attack through the PD-1 and PD-L1 pathway, with PD-L1 expressed in higher numbers on the tumor cells and PD-1 expressed on the activated T, NK, and B lymphocytes, macrophages, dendritic cells (DCs), and monocytes [12]. Several studies have shown this association, thus explaining the evasion of tumor cells from the immune attacks by the body facilitating their growth [13,14]. Antibodies to PD-L1 are now accepted modes of immunotherapy for cancers. [15,16]

Pregnancy is a physiologically altered state of the female body carrying the fetus, which is immunologically alien to the maternal system as it expresses autogenic maternal antigens along with allogenic paternal antigens [17]. Yet, the fetus is protected from maternal immune attacks by some mechanisms. PD-1/PD-L1 is proposed to be one of the mechanisms that provide immunotolerance to the growing fetus [18,19,20]. Studies have revealed a high expression of PD-L1 in syncytiotrophoblasts of the placenta, pointing to its role in the protection of the fetus from maternal immunological attacks [21].

sPD-L1 is the fraction of PD-L1 detected in serum. Our study showed a higher level of sPD-L1 in normal pregnant women and pregnant women with preeclampsia (both in groups 2 and 3) compared to non-pregnant women (group 1), though the difference was not statistically significant (Table 1). The difference between groups 3 and 2 was also less compared to groups 1 and 2. Other studies too have shown a rise in sPD-L1 in pregnancy. Okuyama et al., in their study, observed an elevated sPD-L1 level in pregnant women compared to cord blood levels and non-pregnant women [5]. Pawel et al. also documented a high value of sPD-L1 in pregnancy, which increased during the course of the pregnancy, with the highest value registered in the third trimester [22]. Few studies have shown a significant difference in PD-L1 in preeclampsia [23,24]. Meggyes et al. observed a rise in PD-1/PD-L1 expression on the cell surface of effector cells, but the upregulation of the PD-1/PD-L1 pathway is associated with the lack of its activity in preeclampsia [23]. This is in accordance with our study, though we did not find a statistically significant difference, which may be attributed to the small sample size of our study. Mittelberger et al. documented the downregulation of PD-L1 expression on maternal macrophages in the decidua of preeclamptic patients, leading to inflammation observed in preeclampsia [24]. Miko et al. observed that the blockade of PD-L1 in murine pregnancies leads to an increased fetal resorption rate associated with decreased fetal survival [25]. Immunological dysregulation may play a role in the yet-to-be-established pathogenesis of preeclampsia. To establish a definite link, a further study on a larger sample size is required in this scenario.

Tregs are defined as a subset of T cells with identifying markers CD4+CD25+FOXP3. Tregs play an important role in countering autoimmunity and protecting the body from autoimmune disorders [26,27]. As depicted in some studies, the growing fetus expresses autogenic maternal and allogenic paternal antigens, and the fetus remains at risk for immunological attack by the maternal system [17,28]. The Tregs are found to play an important role in protecting the fetus from immune attacks by the maternal immune system [29]. Tregs are found in increased percentage in pregnancy compared to non-pregnant fertile women in the same age group [30]. Various studies have proved lower counts of Tregs in preeclampsia resulting in fetal growth restriction and fetal loss [31,32]. In our study, we observed a significant decrease in Tregs in preeclampsia women (group 3) compared to normal pregnant women and non-pregnant women (groups 1 and 2) (Table 1). There was a significant rise in Tregs in normal pregnant women compared to non-pregnant women in the same age group. This observation indicates the protective role of Tregs in pregnancy, which is disturbed and deranged in preeclampsia. Our observation is corroborated by a few other studies done [33,34,35].

There exists a fine balance between Tregs and Th17 immunostimulatory cells. The ratio between Tregs/Th17 determines the immunoprotection and immunotolerance in our body and plays an important role in preeclampsia [36,37]. Tian et al. have shown in their study that PD-1/PD-L1 stimulates the differentiation of Tregs and inhibits the formation of Th17 cells, thus creating a niche of immunotolerance [38]. They documented in their findings that “treating preeclampsia like rat models with the protein PD-L1-Fc leads to a reverse of the Treg/Th17 imbalance and thereby to a protective effect on mother and fetus.” Few other studies have also substantiated this novel role of PD-1/PD-L1 pathway [39,40].

In our study, there was a rise in sPD-L1 in the serum of the preeclampsia group compared to normal pregnancy and non-pregnant women. The rise was also seen in normal pregnant women, with the lowest value observed in non-pregnant women. As discussed earlier, this indicates the role of the PD-L1/PD-1 pathway in providing immunotolerance to the fetus.

Our study also depicted a rise in Tregs in normal pregnant women compared to the non-pregnant group, which suggests the role of sPD-L1 in facilitating the differentiation of Tregs. There was a fall in Tregs in the preeclampsia group, though a rise in sPD-L1 was seen, thus having a negative correlation of sPD-L1 in group 3 consisting of preeclampsia women (Table 2). This may be due to an unresponsiveness of the immune system in the differentiation of Tregs under the influence of the PD-L1/PD-1 pathway in preeclampsia, thus creating immunological derangement, which may play a role in the pathogenesis of the disease. This hypothesis was also suggested by Meggyes et al. [23]. Daraei et al. found a decrease in Tregs and increased expression of PD-1 on the Tregs in preeclampsia and postulated it to be Tregs “exhaustion” associated with its reduced function and attributing to the pathogenesis of preeclampsia [41]. Zhang et al. documented a decrease in PD-L1/PD-1 expression in the placenta with an imbalance in the ratio of Tregs/Th17 in preeclampsia and an increased frequency of Th17 cells. In our study, too, the rise in sPD-L1 in preeclampsia cases was not significant in spite of a drastic significant decrease in Tregs, which corroborates with the findings of Zhang et al. [9]. Administration of PD-L1 Fc led to increased differentiation of peripheral naïve CD4+ T cells into Tregs [9]. The establishment of this link needs more elaborate experiments and studies with a bigger sample size and cell analysis in detail.

Antibodies against PD-1/PD-L1 showed increased pregnancy loss [42], whereas PD-L1 Fc administration has been proven to have a protective effect, both on the mother and fetus, in vivo by stimulating the PD-1/PD-L1 T cell co-inhibitory pathway and eventually reversing Tregs/Th17 imbalance [38]. The negative correlation observed between sPD-L1 and Tregs in the preeclampsia women in our study thus may help further studies and trials on PD-L1 Fc administration as a novel therapeutic approach in preeclampsia [9].

TGF-β1, a key inducer of peripheral Tregs, and thymus maturation are both requirements for natural Tregs. Literature data suggest that TGF-β1 promotes the differentiation of CD4+T cells into Tregs initially, and then TGF-β1 is secreted by Tregs, which maintains the inhibitory effects of Tregs by activating its receptors. Antibodies against TGF-β1 or defective TGF-β1 expression lead to weakening or loss of Treg inhibitory activity. According to studies, preeclampsia patients have higher amounts of TGF-β1 in their decidua than healthy controls do. Increased levels of TGF-β1 prevent the particular subgroups of decidual NK (dNK) cells from activating, which contributes to the development of preeclampsia [43]. Our study revealed a high level of TGF- β1in the preeclampsia group compared to normal pregnancy and normal fertile women (Table 1). Studies document varied opinions on TGF-β1. Few studies document higher levels of preeclampsia [44,45,46], whereas some others showed a lower value of preeclampsia compared to normal pregnancy [47]. Few others found no difference between preeclampsia and normal pregnancy in TGF-β1 levels [48,49]. These varied results suggest that pregnancy is a condition associated with higher levels of anti-angiogenic and pro-inflammatory factors than the non-pregnant state and that preeclampsia is associated with an imbalance of these factors in the maternal circulation [50]. A positive correlation was observed between TGF-β1 and sPD-L1 in our study (Table 2). The significant rise in TGF-β1 level in preeclampsia cases associated with a significant decrease in Tregs again points toward the “exhaustion” of Tregs [41] and its unresponsiveness toward the stimulus provided by TGF-β1 and PD-1/PD-L1 pathway resulting in an immunological derangement in PE.

Our study also revealed a significant increase in inflammatory acute phase reactants like hsCRP and inflammatory cytokine IL-6 in the preeclampsia group (group 3) compared to normal pregnant women (group 2) and non-pregnant women (group 3) (Table 1). This indicates an ongoing inflammation in preeclampsia. Tregs suppresses inflammation and provides immunotolerance [51]. Derangement in the PD-1/PD-L1 and Tregs association may be the contributing factor toward the inflammation observed in preeclampsia. Treatment with low-dose IL-2 therapy has been shown to increase the Tregs population and proven to be clinically effective in a few trials on type 1 diabetes mellitus, graft versus host disease, systemic lupus erythematosus (SLE), and rheumatoid arthritis [52,53,54,55]. Hence, the expansion of Tregs with IL-2 and TGF-β may be potential therapeutic targets for the management of preeclampsia along with the above-suggested PD-L1 Fc. This has been documented by other studies [56,57,58].

A positive correlation was observed between IL-6, hsCRP, and sPD-L1 (Table 2), which may be explained as an attempt to stimulate the expression of PD-1/PD-L1 by the ongoing inflammation present in preeclampsia due to immunological dysregulation and imbalance between PD-1/PD-L1 and Tregs/Th17 ratio [59,60,61].

Serum albumin showed a negative correlation with sPD-L1 (Table 2). Albumin is a negative acute phase reactant. In the scenario of inflammation and proteinuria associated with preeclampsia, serum albumin is expected to decrease, whereas sPD-L1 increases as a protective effect. However, not many studies have been done on this, and further confirmation is needed.

This study had a few limitations. The sample size of the study was small. A larger sample size can give more definitive information regarding the association between sPD-L1 and Tregs alteration in preeclampsia pathogenesis. Further, it is a cross-sectional study. A prospective follow-up study could have been more informative in the prediction and prevention of the disease. A study of the PD-L1/PD-1 pathway and Tregs at the cellular level has not been done. This can be a part of futuristic replicative studies on this aspect of preeclampsia.

## Conclusions

Our study shows an increase in sPD-L1 and TGF-β1 and a decrease in Tregs with an increase in inflammatory markers like IL-6 and hsCRP levels in preeclampsia have a potential implication for early diagnosis and management of the condition. sPD-L1 can be used as a marker to predict impending preeclampsia by measuring its levels trimester-wise along with counts of Tregs. PD-L1 and Tregs can be potential therapeutic target molecules for early management of preeclampsia. Stimulation of expansion of Tregs with IL-2 and TGF-β1 resulting in decreased Tregs/Th17 ratio along with PD-L1 Fc administration can be a novel therapeutic approach for managing preeclampsia.
